# Regional scale diversity and distribution of soil inhabiting *Tetracladium*

**DOI:** 10.1186/s40793-024-00646-6

**Published:** 2024-12-18

**Authors:** Anna Lazar, Robert I. Griffiths, Tim Goodall, Lisa R. Norton, Ryan M. Mushinski, Gary D. Bending

**Affiliations:** 1 School of Life Sciences, The University of Warwick, Coventry, CV4 7AL UK; 2 School of Natural Sciences, Bangor University, Bangor, LL57 2UW UK; 3 UK Centre for Ecology & Hydrology, Lancaster Environment Centre, Library Avenue, Bailrigg, Lancaster, LA1 4AP UK

## Abstract

The genus *Tetracladium* has historically been regarded as an aquatic hyphomycete. However, sequencing of terrestrial ecosystems has shown that *Tetracladium* species might also be terrestrial soil and plant-inhabiting fungi. The diversity of *Tetracladium* species, their distribution across ecosystems, and the factors that shape community composition remain largely unknown. Using internal transcribed spacer (ITS) amplicon sequencing, we investigated the spatial distribution of *Tetracladium* in 970 soil samples representing the major ecosystems found across the British landscape. Species of the genus were found in 57% of the samples and across all vegetation types. The *Tetracladium* sequences we recovered included species common in aquatic ecosystems. However, we found five additional clades that clustered with environmental sequences previously found in terrestrial environments. The community composition of the *Tetracladium* OTUs was mainly related to vegetation type and soil pH. Strikingly, both taxon richness and overall abundance were highest in arable soils and showed positive relationships with soil pH. *T. maxilliforme* and a taxon of environmental sequences, *Tetracladium* group 1, was the biggest group, had the most relative abundance across ecosystems and was found in all vegetation types. Overall, this study provides insights into the community composition patterns of *Tetracladium* in terrestrial ecosystems and highlights the importance of vegetation characteristics in shaping *Tetracladium* communities.

**Supplementary Information:**

The online version contains supplementary material available at 10.1186/s40793-024-00646-6.

## Introduction

Aquatic hyphomycetes are a group of phylogenetically diverse fungi which grow on decaying leaves and plant litter in streams [1]. These fungi do not share common morphological characteristics except for their conidiospores (e.g. sigmoid or tetraradiate), which are considered to be an adaptation to aid dispersal in flowing systems [2]. As high-throughput sequencing techniques have become more widely accessible, some aquatic hyphomycetes have been found in the soil and as plant-colonising endophytes in a range of terrestrial environments [3].

The genus *Tetracladium* is an aquatic hyphomycete that is commonly found around the world [4–6]. Their spores have been detected in freshwater systems but also in the water film covering fallen forest litter [7–9]. After the turn of the century, widespread use of environmental metabarcoding to determine the composition of fungal communities showed that *Tetracladium* sequences were common in terrestrial systems. However, there are very few isolates to support the currently described species, most of which are described from aquatic environments. It has been hypothesised that there was under-reporting of *Tetracladium* species in terrestrial habitats before the 2000s because of the nature of finding a supposed aquatic organism in a terrestrial environment [10]. It is not yet known whether the aquatic species described based on spore morphology and the environmental DNA sequences from terrestrial habitats belong to the same organisms. However, some species may have diverse ecological functions, as nuclear ribosomal internal transcribed spacer (ITS) amplicon analysis has shown no sequence-based differences between aquatic and terrestrial strains [10, 11]. Species of the genus were initially observed as an endophyte in riparian plant roots [12], before being found more broadly within terrestrial habitats in the roots of Equisetaceae [13, 14], Bryophytes [15–18], monocot species within Asparagales [3, 19, 20], Liliales [21], and Poales [22–24], as well as dicot species within Ericales [25], Brassicales [26, 27] and Vitales [28], showing no apparent host preference. Most studies that have reported soil and root inhabiting *Tetracladium* species are from farmed habitats, although unmanaged habitats such as woodlands are under-represented in the studies [29–31].

The terrestrial and aquatic ecology of the genus and the extent to which these lifestyles are linked is still unknown. Selosse et al. [10] suggested that the endophytic nature of aquatic hyphomycetes is an adaptation by the fungi to build their biomass before abscission so they are already occupying the niche, ready to decompose plant litter when it reaches freshwater. Consequently, *Tetracladium* species should have a higher abundance in aerial plant tissues compared to the roots, although there is currently no evidence to suggest that this is true. However, Anderson and Shearer [32] showed that *T. marchalianum* maintained high genotypic diversity throughout the year, indicating that their endophytic lifestyle could serve as a genetic reservoir for the population. Importantly, a landscape scale study showed that root-inhabiting *Tetracladium* species had a co-exclusion relationship with root pathogenic fungi in oilseed rape crops [33] and the relative abundance of *Tetracladium* species within roots was positively correlated with crop yield [11, 33] suggesting that *Tetracladium* species are signatures of a healthy microbiome and potentially endophytes which benefit plant health.

In a previous study, we investigated the landscape diversity of *Tetracladium* and drivers of community composition in agricultural soil, finding that endophytic colonisation was related to soil pH, phosphorus concentration, and crop rotation [11]. The relevance of this work to broader landscapes and diverse habitat types remains unclear. Here we build on the earlier study to investigate the factors driving the diversity and distribution of *Tetracladium* across the broader landscape, encompassing both natural and managed vegetation types. Using soils from the Countryside Survey national monitoring scheme, a long-term, large-scale survey sampling of vegetation types and soil characteristics across Great Britain, we investigated [1] the diversity of soil-inhabiting *Tetracladium* [2] the communities of *Tetracladium* inhabiting soil across different habitats and [3] the vegetation characteristics, climatic variables and soil chemical and physical properties which determine the diversity and distribution of *Tetracladium* at the regional scale.

## Materials and methods

### Sample collection and analyses

Soil cores were collected in 2007 from 233 1 km^2^ squares across the UK as part of the Countryside Survey (http://www.countrysidesurvey.org.uk/). Within each square, five soil cores were sampled (5 cm diameter, 15 cm deep) from the centre of randomly allocated 200 m^2^ sub-plots. For some, lower numbers of samples were collected because of access limitations. The soil samples were kept separate and stored frozen at -20 °C. The sampling details are described in Griffiths et al. [34]. Soil chemical and physical characteristics (pH, total carbon, total nitrogen, organic carbon, total phosphorus content) and the Ellenberg N (nitrogen) metric for the vegetation plot at which soil samples were located were determined. Ellenberg values relate to the suite of plant species in the plots where the soils are sampled, with Ellenberg N values related to the extent to which plant species perform well or otherwise in high nitrogen conditions [see full details in CS Technical Report No. 3/07 [35]]. Field measurements of flora were recorded at each sampling site; then plots were categorised into aggregate vegetation types after sampling [36]. Aggregate vegetation class was assigned based on plant species present using the Countryside Vegetation System, a vegetation classification specially designed for the Countryside Survey [36, 37]. Short descriptions of aggregate vegetation classes are provided in Supplementary Table 1 and Supplementary Fig. 1, and detailed descriptions can be found at https://nora.nerc.ac.uk/id/eprint/4311/. Samples with missing metadata (195) were disregarded for this study.

DNA was extracted from 0.2 g of soil using the PowerSoil-htp 96 Well DNA Isolation kit (Qiagen, Hiden, Germany) according to the manufacturer's protocols. Fungal internal transcribed spacer 2 (ITS) amplicon sequences were generated using a 2-step amplification approach using primers fITS7 (5’-GTGARTCATCGAATCTTTG-3’) [38] and ITS4 (5’-TCCTCCGCTTATTGATATGC-3’) [39]. Standard negative control PCR reactions were performed, and the use of dual indexing eliminated issues of tag swapping as unexpected combinations were assigned as undetermined in downstream processing. Illumina Miseq sequencing was performed as described previously [40]. Sequences were processed in R [41] using DADA2 [42]. The amplicon reads underwent pre-processing using *cutadapt* [43] to eliminate primer sequences and mitigate read-through concerns. Subsequently, reads were truncated to 205 nucleotides for the forward strand and 160 nucleotides for the reverse strand. Sequences exhibiting Ns and errors surpassing a maximum expected error threshold of 5 were filtered out. Denoising, merging, chimera detection, and taxonomic assignment were performed using default parameters. Taxonomic assignments were made employing the Unite v7.2 database [44]. Taxonomic classification was carried out using the Naive Bayesian Classifier [283] with a kmer size of 8, 100 bootstrap replicates and a minimum bootstrap confidence of 50 for assigning a taxonomic level. Sequences were clustered to operational taxonomic units (OTUs) [45] at a 97% minimum identity threshold using the PIPITS pipeline [46] and those OTUs assigned as *Tetracladium* were selected for use in the current study.

### Phylogenetic analyses

For analysis of the phylogeny of the *Tetracladium* sequences, the most closely related sequences to these OTUs were accessed from the NCBI GenBank, including two representative ITS2 sequences from all described species (Suppl. Table 2). Sequences were aligned with the OTU sequences using MAFFT v.7 (e-ins-I algorithm) [47]. To build a phylogenetic tree, maximum likelihood analyses were performed with RAxML on the CIPRES Science Gateway using the default setting with 1000 bootstrap replicates [48, 49].

### Statistical analyses

Richness plots with observed species counts were used to study OTU community composition differences across the vegetation types using the Kruskal–Wallis test with Dunn's posthoc test in *vegan* (version 2.6–4) in R (version 4.2.2) [41, 50]. Rarefaction curves were created to assess the extent to which fungal richness was captured. Principal Correspondence Analysis (PCoA) ordination plots were generated based on dissimilarities calculated using the Bray–Curtis index to relate the distribution of *Tetracladium* OTUs to vegetation types. Additionally, non-metric multidimensional scaling (NMDS) ordination plots were generated based on dissimilarities calculated using Raup-Crick dissimilarity to relate the distribution of *Tetracladium* OTUs to vegetation types to account for unequal sampling sizes. Analysis of similarities (ANOSIM) was used to further study community composition differences across vegetation types *(vegan* version 2.6–4 [50]). A heatmap was constructed to examine the distribution of *Tetracladium* OTUs across vegetation types to find unique and commonly occurring *Tetracladium* groups. Permutational Multivariate Analysis of Variance Using Distance Matrices (PERMANOVA) was performed using *anova2* in R to assess the effect of aggregate vegetation class, soil properties, and location on OTU distribution. Significance values were corrected using the false discovery rate with the Benjamini–Hochberg method. Heatmaps, rarefaction analyses, and PERMANOVA were carried out using *phyloseq* (version 1.38.0) [51] in R. Maps were created using *phylogeo* (version 0.99.6.3) [52] in R. Faceted and stacked bar plots showing *Tetracladium* group reads in different aggregate vegetation classes were produced with *ggplot2* (version 3.3.6) [53]. To understand the drivers of community structure and OTU relative abundance (relative to the whole fungal community), variation partitioning (VP) was performed to determine the co-variance of the metadata variables. Finally, based on the PERMANOVA and VP results we created piecewise structural equation models (PSEMs). Variation partitioning was performed using *vegan* (version 2.6–2) in R (version 4.12) [50], PSEM was performed using *piecewiseSEM* (version 2.3.0) [54] in R. OTU relative abundance and continuous metadata variables were normalised using Min–Max normalisation.

## Results

### Tetracladium diversity and distribution across vegetation types

**Fig. 1:**
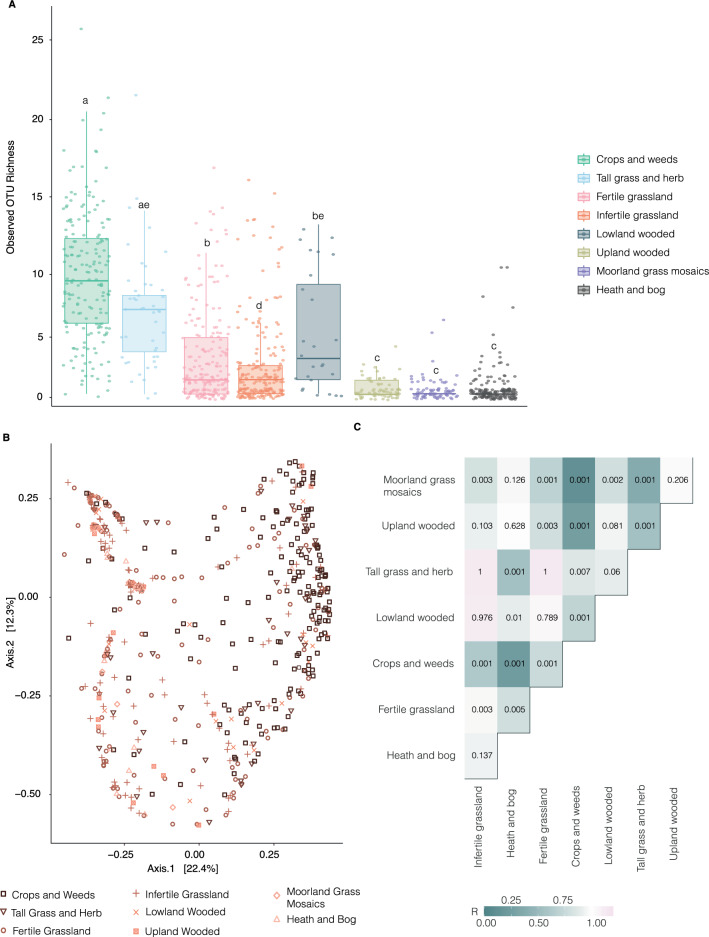
Distribution of diversity across the vegetation types. **A** – Observed OTU richness in the described Vegetation types. Error bars represent ± standard error of the mean. Bars with different letters are significantly different (*P* < 0.05). vegetation types are ordered based on disturbance. **B** – Principal Coordinates Analysis (PCoA) of the *Tetracladium* OTU community estimated by Bray–Curtis similarity of the vegetation types. Vegetation type colour denotes disturbance level (highly disturbed to natural habitats are shaded from dark to light). **C** – Analysis of similarities (ANOSIM) of the samples across the vegetation types. Numbers indicate significance values. The colour indicates ANOSIM statistic R values

Across all samples, the total number of high-quality ITS sequences was 37 801 182 (from 420 276 828 raw reads), out of which 103 219 corresponded to *Tetracladium*. Across the 970 samples, we found 54 OTUs grouped at a 97% similarity level representing *Tetracladium*. Rarefaction curves indicate that the sequencing depth for *Tetracladium* was adequate, demonstrating that the fungal communities were sufficiently captured in the soil samples across the different vegetation types at the applied sequencing depth (Suppl. Figure 2). There was a significant difference in observed *Tetracladium* OTU richness between vegetation types (Fig. 1A). Crops and weeds had the highest average OTU richness (*P* < 0.05) followed by tall grass and herb and lowland wooded. Fertile and infertile grassland had significantly lower average OTU richness (*P* < 0.05), while heath and bog, moorland grass mosaics, and upland wooded had close to zero average OTU richness (Fig. 1A). The clustering of *Tetracladium* OTUs in the samples based on vegetation type was visualised using PCoA ordination plots (Fig. 1B). Samples from the crops and weeds vegetation type had similar *Tetracladium* communities and formed a distinct cluster in the ordination plot (Fig. 1B). Based on Raup-Crick dissimilarity NMDS ordination, most of the samples had similar *Tetracladium* communities to each other and formed a cluster in the ordination plot (Suppl. Figure 3). Samples from grassland vegetation types (fertile grassland, infertile grassland, and moorland grass mosaics) formed clusters during ordination, however, they were not different based on vegetation types. The *Tetracladium* OTU community composition was different between vegetation types based on ANOSIM (R = 0.237, *P* = 0.001). Communities inhabiting crops and weeds were significantly different to those from all other vegetation types (*P* = 0.001 for all except for tall grass and herb, where *P* = 0.007). The composition of *Tetracladium* communities in tall grass and herbs was significantly different from those in heath and bog (R = 0.448, *P* = 0.001), upland wooded (R = 0.449,* P* = 0.001), and moorland grass mosaics (R = 0.620, *P* = 0.001). Communities inhabiting lowland wooded, fertile grassland and heath and bog samples were generally not different from samples from other vegetation types (Fig. 1C).

**Fig. 2:**
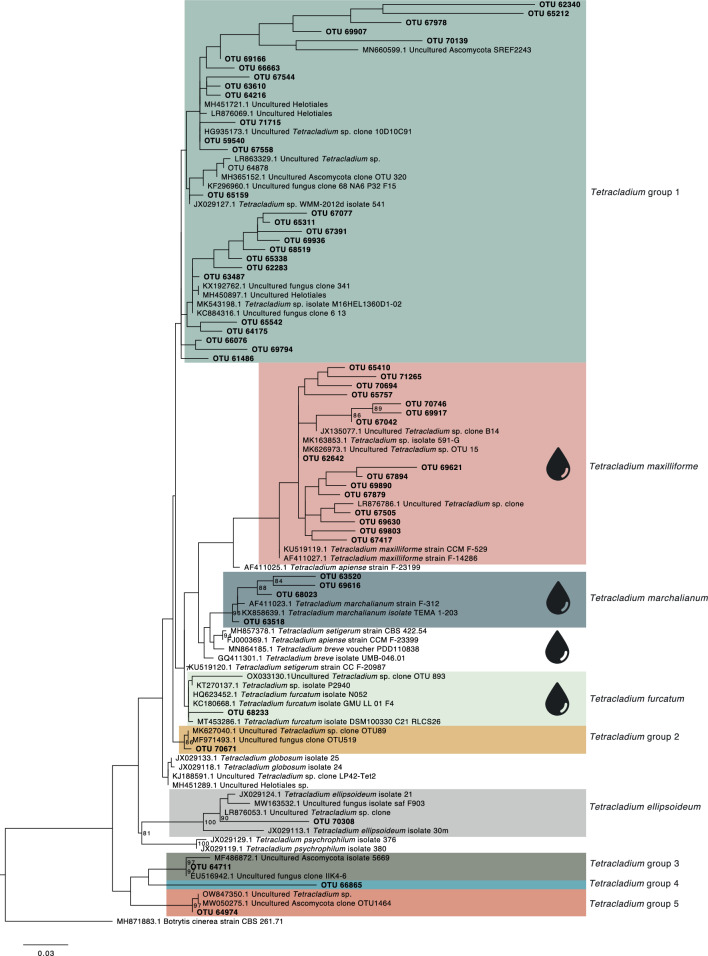
Internal transcribed spacer 2 sequence-based maximum likelihood tree with posterior probability values of the *Tetracladium* OTUs, reference sequences and *Botrytis cinerea* as an outgroup. The scale bar denotes the number of nucleotide differences per site. Taxa with water droplets next to them are traditionally considered aquatic

**Fig. 3:**
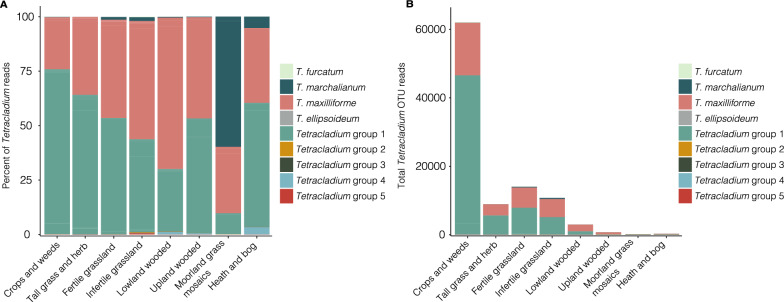
Stacked bar plots showing **A** – the proportion of reads for *Tetracladium* groups, **B** – absolute read numbers of *Tetracladium* group reads across vegetation types

Based on the maximum likelihood phylogenetic tree, we assigned the OTUs to nine groups (Fig. 2). Groups 1 – 5 represented *Tetracladium* groups to which the closest sequence matches were from environmental samples from terrestrial soil or roots (Suppl. Table 2). The rest of the OTUs represented the aquatic species *T. maxilliforme*, *T. marchalianum*, *T. furcatum*, and *T. ellipsoideum*. Most of the OTUs clustered into three groups, *Tetracladium* group 1, *T. maxilliforme*, and *T. marchalianum* with 50%, 29.6%, and 7.4% of the OTUs, respectively. The remaining groups had a single OTU (Fig. 2). The abundance and distribution of these groups through the vegetation types were variable. Analysis of the distribution of individual OTUs across the samples and the vegetation types indicated the presence of a core *Tetracladium* OTU group that was present in most vegetation types (six or more) and samples (Suppl. Figure 4 and 5). OTUs 59540 which clustered in *Tetracladium* group 1, and 67042 and 62642 which clustered with *T. maxilliforme*, were found in all vegetation types and were present in most of the samples where *Tetracladium* sequences were found. These OTUs generally had the highest abundance in the whole dataset, representing 40% of OTU reads. The combined relative abundance of the *Tetracladium* OTUs compared to all fungal OTUs was 5.8% in some of the crops and weeds sites (Suppl. Figure 5). 38% of the OTUs were found in 3–5 vegetation types, while generally lower abundant OTUs (OTUs 62340, 63520, 64974, 66865, 69616, 69803, 69907, 69917, 70746, 70139, 71256), were found in one or two vegetation types, and combined represented 22% of relative abundance (Suppl. Figure 5). *T. maxilliforme* and *Tetracladium* group 1 dominated the sequence reads from most of the vegetation types except for moorland grass mosaics where *T. marchalianum* had the most reads (Fig. 3A). Considering the total number of *Tetracladium* reads, species of the genus were the most abundant in crops and weeds, followed by the grasslands (fertile, infertile, and tall grass and herb), then lowland wooded (Fig. 3B). Altogether *Tetracladium* group 1 was the most abundant group throughout the dataset followed by *T. maxilliforme* (Fig. 3).

### Drivers of distribution and community composition

We related the collected soil metadata to *Tetracladium* OTU diversity across all the samples using PERMANOVA. We found that vegetation type, pH, longitude, Ellenberg N, soil moisture content, total nitrogen, and latitude were significant drivers of community composition. Vegetation type and soil pH were the most important factors explaining 10.66% and 4.91% of variation respectively (*P* = 0.001 for both), with the other factors contributing between 0.3% and 0.55% of the variation (Table 1). To visualise the relationships between location and the vegetation type, pH, and moisture content of the sampling sites maps were created with combined relative abundance percentages of the *Tetracladium* OTUs (Suppl. Figure 6). Visual assessment indicated that higher relative abundance sites were found in the south and the east of Great Britain showing higher occurrences in the crops and weeds and tall grass and herb vegetation types (Suppl. Figure 6A). Higher relative abundance of *Tetracladium* OTUs was apparent at higher pH and lower moisture content locations (Suppl. Figure 6B and C).

**Table 1:** Permutational Multivariate Analysis of Variance (PERMANOVA) of the percent variation of the *Tetracladium* OTUs explained by soil physicochemical properties and vegetation type

	Degrees of freedom	Sum of squares	R2	F	P adjusted
Habitat	7	20.3690	0.1066	9.2453	**0.0099**
pH	1	9.3850	0.0491	29.8169	**0.0099**
Longitude	1	0.8520	0.0045	2.7062	**0.0198**
Ellenberg N	1	0.7220	0.0038	2.2945	**0.0198**
Moisture	1	0.5810	0.0030	1.8450	**0.0495**
Total N	1	1.0550	0.0055	3.3510	**0.0099**
Organic Carbon	1	0.4740	0.0025	1.5070	0.1782
Total C	1	0.4200	0.0022	1.3358	0.2376
Latitude	1	0.5710	0.0030	1.8154	**0.0495**
Total P	1	0.2240	0.0012	0.7123	0.7030
Residual	497	156.4250	0.8186		
Total	513	191.0780	1.0000		

Significant *P* values are highlighted in bold

**Fig. 4:**
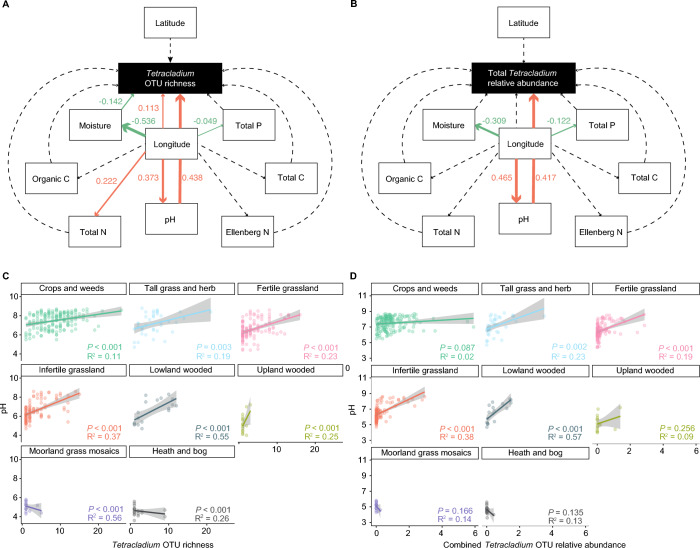
Drivers of observed diversity and relative abundance of *Tetracladium* OTUs. Piecewise structural equation model (PSEM) with **A** – observed OTU richness, and **B** – OTU relative abundance as response variables and vegetation type as the random variable. Path diagram of the PSEM showing direct and indirect effects with standard estimates for linear mixed effects correlations. Dotted lines indicate non-significance in the model. Arrow sizes indicate standard effect size, arrow colours indicate a positive or a negative relationship (orange – positive, green – negative). OTU relative abundances and pH are normalised. Linear regression line fitted between *Tetracladium* OTU **C** – diversity, **D** – relative abundance, and pH with significance values across the vegetation types. OTU relative abundances and pH are normalised. The shaded region represents the 95% confidence limit for the estimated prediction. Model indicators and coefficients for the PSEMs can be found in Suppl. Table 3

As the maps indicated relationships between location, vegetation type, and soil properties we conducted variance partitioning (VP) analyses to estimate the importance of constraining variables along short gradients. We found that location alone (latitude and longitude) did not explain any variation in *Tetracladium* communities (Suppl. Figure 7). Vegetation type explained a lower percentage of variation on its own than soil properties (2%, and 5%, respectively), however, there was co-variation between them, and this accounted for 5% of the total variation. There was a combined effect of soil properties, location, and vegetation type of 3%. Finally, to further test the effects of soil nutrients, location and vegetation type on OTU abundance and diversity, we created piecewise structural equation models (PSEMs) with vegetation type as the random variable, soil pH, soil moisture content, total nitrogen, total carbon, total phosphorus, Ellenberg nitrogen, organic carbon, longitude, and latitude as the fixed variables, and *Tetracladium* OTU richness or total relative abundance as the response variable. Then we included location as a response variable to the above-mentioned soil property variables with vegetation type as a fixed variable. We found a strong correlation between both OTU richness (Fig. 4A, Suppl. Table 3) and relative abundance (Fig. 4B, Suppl. Table 3) with pH (*P* < 0.001 for both). Observed richness had a positive correlation with longitude (*P* = 0.002) and a negative correlation with soil moisture content (*P* = 0.041). Total P, soil moisture, pH, and total N had significant correlations with longitude (*P* = 0.049, < 0.001, < 0.001, 0.001, respectively) thus indirectly affecting observed richness. Furthermore, pH, soil moisture, and total P also had a significant correlation with longitude (*P* < 0.001 for all) in the relative abundance model, even though longitude was not a significant factor in shaping the relative abundance. As pH was the most important factor in both models, we created scatter plots with trendlines fitted to the OTU richness (Fig. 4C) or relative abundance (Fig. 4D) to better understand the effect of soil pH in the different vegetation types. Soil pH had a significant correlation with OTU diversity in the crops and weeds, tall grass and herbs, fertile grassland, infertile grassland, lowland wooded, and upland wooded vegetation types (Fig. 4C). *Tetracladium* OTU relative abundance was significantly correlated with pH in the tall grass and herbs, fertile grassland, infertile grassland, and lowland wooded vegetation types.

## Discussion

In this study, we investigated *Tetracladium* community composition and the factors that shape their occurrence in soils on a regional scale, across various temperate vegetation types. *Tetracladium* was widely distributed, occurring in 554 out of the 970 samples (57%) with varying abundance. There was a significantly higher OTU richness in agricultural sites, grasslands, and lowland woodlands than in other vegetation types. We found 54 OTUs that represented *Tetracladium*. Fifty-nine percent of the OTUs did not cluster with any known species and only corresponded to environmental sequences. The rest of the OTUs, except for one, were clustered with taxa traditionally considered aquatic. *Tetracladium* group 1 and *Tetracladium maxilliforme* were the most abundant groups in all vegetation types except for moorland. A core group of *Tetracladium* OTUs was identified in most samples and vegetation types. We found vegetation type, location, and soil physical and chemical properties to be drivers of the community composition of the *Tetracladium* OTUs. Finally, structural equation modelling revealed that pH had a positive relationship with community composition when vegetation type was considered a random effect.

### Tetracladium is a common part of the soil microbiome

The genus *Tetracladium,* although traditionally considered aquatic, has been found in soils across the world. In a previous study, we found species of the genus to be an abundant member of the oilseed rape microbiome in all soil compartments (bulk soil, rhizosphere, and roots) on a landscape scale in the UK [11]. Here, using a dataset with more comprehensive coverage of terrestrial vegetation types, we detected *Tetracladium* in all sampled vegetation types across Great Britain. While *Tetracladium* has been found globally [13, 24, 25, 28] this is the first time its distribution has been studied systematically on a large geographic scale across multiple different vegetation types.

*T. apiense, T. ellipsoideum*, *T. maxilliforme, T. marchalianum* and *T. furcatum* have been found several times in soil from various habitats including tundra [55], temperate forest [25], agriculture [29], and disturbed grassland [26] although *T. maxilliforme*, *T. furcatum*, and *T. marchalianum* were historically described as aquatic species [1, 56, 57]. We found a group of OTUs that were present in most of the samples and vegetation types, comprising a core *Tetracladium* pool. There is little previous knowledge about the niche preference of *Tetracladium* although it has been found in the soil of various vegetation types and in plant species as endophytes with no evidence for host preference. Compartment preferences for most *Tetracladium* species have not been determined but evidence suggests that they may have either root or soil preferences. In our previous work, *T. furcatum* and *T. maxiliforme* were recruited into roots and showed higher abundance there relative to bulk soil, while the reverse was true for a variety of uncharacterised *Tetracladium* OTUs [11].

In this study, we found many OTUs that correspond with aquatic *Tetracladium* species but most of the OTUs clustered in groups comprising environmental sequences. Most studies describing fungal diversity from environmental samples report *Tetracladium* sequences without identifying them to the species level, and an increasing number of these genus-level sequences are being released to accessible databases [23, 58, 59]. This study highlights the need for determining the phylogenetic and evolutionary relationships of the genus. However, the ITS region is heterogeneous within the genus due to the multicopy nature inherent in ribosomal genes [60]. Consequently, the ITS region is acknowledged not as a definitive taxonomic tool but rather as a facet within the broader taxonomic characterization. Therefore, further analyses are needed such as genome level comparison to accurately capture the species diversity of the genus. We found *Tetracladium* OTUs in 57% of the samples even if it was infrequent in some cases. Therefore, we can conclude that *Tetracladium* is a common part of the soil mycobiome across vegetation types on a regional scale with undiscovered and undescribed diversity and ecosystem functions.

This study focused on fungal ITS sequences taxonomically classified as *Tetracladium* using the UNITE database. We then used reference sequences from the GenBank to construct phylogenetic trees. OTUs were queried against the GenBank database using BLASTn, and the top two matches based on sequence identity percentage were selected. The resulting phylogenetic tree exhibited branches corresponding to environmental sequences of *Tetracladium* or unidentified fungal sequences. It is estimated that approximately 3% of metazoan sequences in GenBank are misannotated at the genus level, with the error rate increasing at more specific taxonomic levels [284]. Consequently, environmental sequences annotated as *Tetracladium* may have been incorrectly identified, possibly due to reliance on other environmental sequences lacking associated culture data. Given the complexity of tracing the origins of these annotations, it remains challenging to assess their accuracy fully. Thus, caution is warranted when interpreting the results of this study, and the inherent limitations of sequence-based research must be acknowledged. One potential solution to these challenges is the use of UNITE Species Hypotheses (SH), which offer a standardized method for delimiting, identifying, and working with DNA-based sequences. This approach clusters sequences based on molecular data rather than traditional taxonomic labels. While this methodology enhances the reliability of sequence clustering, it also presents complications, particularly for poorly studied taxa like *Tetracladium*.

### Vegetation type and pH are the main drivers of community composition of Tetracladium OTUs

The complexity of factors shaping the general fungal community of soils is poorly understood [61], and currently, there is also limited understanding of the drivers of *Tetracladium* community composition in the soil. In our previous study of *Tetracladium* in soil and roots of oilseed rape crops, we found a correlation between *Tetracladium* OTU relative abundance in roots with pH, soil nutrients, and oilseed rape rotation frequency [11]. In the current study, we demonstrate the importance of pH and vegetation type for determining *Tetracladium* OTU community composition. We found a positive linear correlation between pH and *Tetracladium* OTU community composition and assembly on a regional scale. Contrary to our findings with *Tetracladium*, saprotrophic fungal communities in soil show higher diversity in acidic soils on a global scale [62]. Saprotrophic fungi release enzymes with pH-dependent activity to break down organic matter [63]. Acidic soils tend to have higher enzyme activity compared to alkaline soils, however, some extracellular enzymes including microbial peroxidases and aminopeptidases show the reverse relationship [64].

The functional significance of *Tetracladium* in terrestrial ecosystems is unclear, particularly the extent to which terrestrial *Tetracladium* are saprotrophs as aquatic members of the genus are. We found vegetation type to have a strong influence on community composition, and although soil organic matter content was not a determinant of community composition the characteristics of organic matter found across these vegetation types vary markedly [65]. Importantly, the heath bog vegetation type had low OTU relative abundance and richness despite including wetland vegetation types. Freshwater *Tetracladium* are typical of flowing water [66, 67], and static, low oxygenated, high polyphenol environments with standing water do not support high abundance or diversity of *Tetracladium* OTUs, despite their high organic matter contents. Similarly, low *Tetracladium* abundance and diversity were found in upland wooded and moorland grass mosaic vegetation types, which represent further high organic matter vegetation types. Despite a likely saprotrophic mode for many *Tetracladium* species, the availability of organic matter is therefore not a key determinant of their distribution.

*Tetracladium* was found predominantly in the crops and weeds vegetation type. Interestingly abundance and diversity were low in lowland wooded and tall grass and herb vegetation types which are adjacent to cropped vegetation types. Relative to other land uses cropped soils are highly managed so that they have high pH, are well-drained and nutrient-rich [68]. While we have shown that pH is a key factor associated with *Tetracladium* community composition, other factors characteristic of agricultural vegetation types could also play a role in supporting *Tetracladium* communities. Organic matter in these soils is largely derived from cereal crop debris in the UK, and saprotrophic *Tetracladium* may be specifically adapted to this type of material. Indeed *T. marchalianum* was identified as a coloniser of wheat residues in a decomposition study [69]. Furthermore, cropped soils are highly disturbed through cultivation and soil management practices such as tillage, and this could also be a factor which favours the selection of *Tetracladium*.

Fungal community composition exploration provides an analysis of the relative abundance of sequence reads, and the absolute number of OTU reads is influenced by the overall quantity of fungal DNA present within a sample and the specific proportional representation of the OTU. Consequently, observed fluctuations in *Tetracladium* communities, including variance across ecosystems may reflect differences in the absolute abundance of *Tetracladium* and of other fungi, highlighting the complex nature of analysing fungal community dynamics. The ITS region is widely regarded as the primary genetic marker for fungal community characterization [42]. While ITS sequencing is a powerful tool for fungal community characterization due to its high variability, ease of amplification, and extensive database support it has a number of limitations. High intraspecific variation can complicate the assignment of sequences to specific species. Fungi often possess multiple copies of the ITS region within their genomes, and these copies can exhibit sequence heterogeneity [44]. Furthermore, the presence of multiple operons contributes to this heterogeneity, leading to an inflated number of taxa. PCR amplification of the ITS region can sometimes produce chimeric sequences [45], which can also lead to an inaccurate representation of fungal diversity. Lastly, universal ITS primers may not equally amplify all fungal taxa, which can induce biases in the detected community composition, leading to some fungi being underrepresented or entirely missing [46]. Combining ITS data with other genetic markers can help mitigate these shortcomings and provide a more comprehensive understanding of fungal diversity and ecology.

## Conclusions

*Tetracladium* is a commonly occurring genus in soil with its diversity and relative abundance strongly influenced by vegetation type and soil pH. Further research is needed to determine the full extent of drivers that shape its community structure, identify their ecological significance, and understand the functional roles *Tetracladium* plays within ecosystems.

## Supplementary Information

Supplementary Figure 1. Countryside Survey vegetation plot data classified by TWINSPAN. Cluster analysis of their mean detrended correspondence analysis scores produced eight aggregate vegetation classes. The figure was adapted from Firbank et al. (2003) (36).

Supplementary Figure 2. Sequencing efficacy of the samples for the fungal ITS sequences. Rarefaction curves showing fungal OTU richness.

Supplementary Figure 3. Non-metric multidimensional scaling (NMDS) of the *Tetracladium* OTU community based on Raup-Crick dissimilarity of the vegetation types. Vegetation type colour denotes disturbance level (highly disturbed to natural habitats are shaded from dark to light).

Supplementary Figure 4. Heatmap showing the distribution of *Tetracladium* OTUs across all samples.
Supplementary Figure 5. Distribution of the OTUs across the vegetation types. Heatmap showing the combined relative abundance percent of *Tetracladium* OTUs across the vegetation types. The colours on the right side of the figure indicate groups from Figure 2. Taxa with water droplets next to them are traditionally considered aquatic.
Supplementary Figure 6. Maps showing the location of the sampling sites, their A - vegetation type classifications, B - soil pH, C - soil moisture content (circle colour), and the combined *Tetracladium* OTU relative abundance percent (circle size).
Supplementary Figure 7. Redundancy analysis of the variables shaping OTU richness. Venn diagram of the variation partitioned variable categories.
Supplementary Table 1. Brief description of the aggregate vegetation classes.

## Data Availability

All raw ITS sequence reads are deposited at the European Nucleotide Archive under project accession PRJEB45286. Furthermore, detailed sample and run accessions are provided in the accompanying data submission (Supplementary Table 4).
