# Supplementary material for: Regional scale diversity and distribution of soil inhabiting Tetracladium
Source: Environ Microbiome. 2024 Dec 18;19:111. doi: 10.1186/s40793-024-00646-6 (PMC11657488; doi:10.1186/s40793-024-00646-6)
Supplement: Supplementary file 1 — Supplementary Figure 1. Countryside Survey vegetation plot data classified by TWINSPAN. Cluster analysis of their mean detrended correspondence analysis scores produced eight aggregate vegetation classes. The figure was adapted from Firbank et al. (2003) (36). [file 40793_2024_646_MOESM1_ESM.pdf]

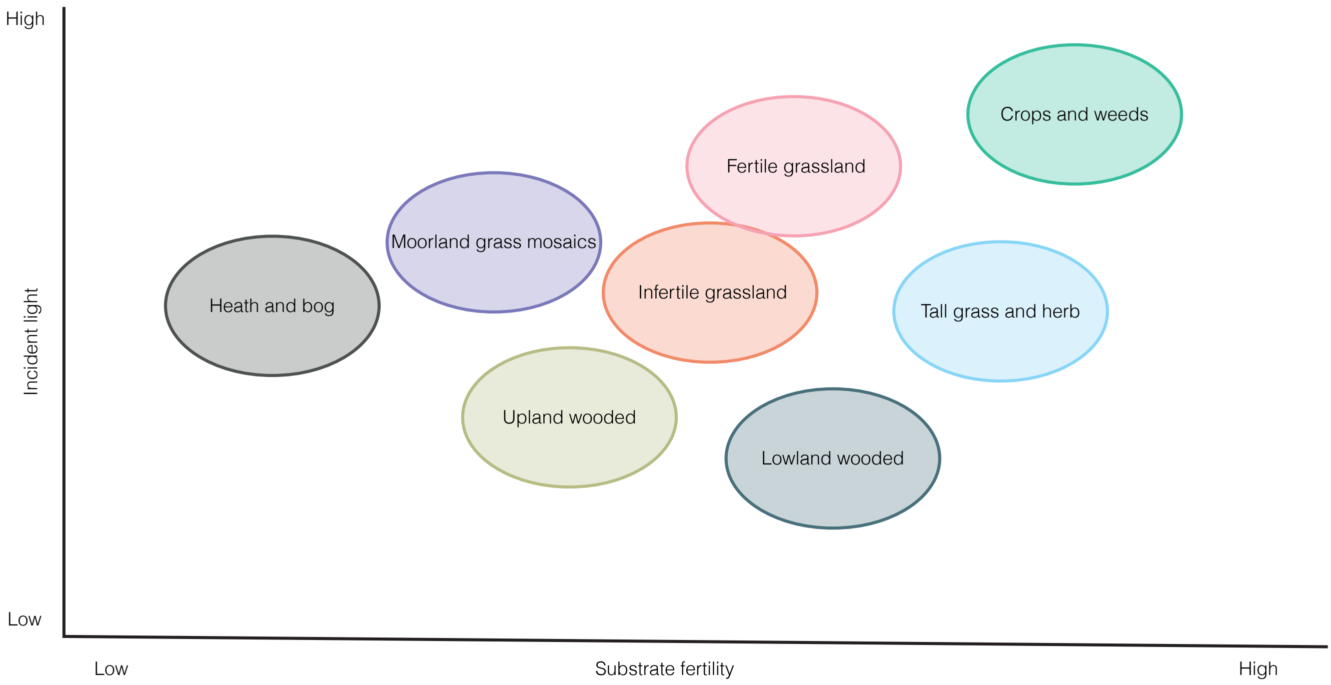

Supplementary Figure 1. Countryside Survey vegetation plot data classified by TWINSPAN. Cluster analysis of their mean detrended correspondence analysis scores produced eight aggregate vegetation classes. The figure was adapted from Firbank et al. (2003) (38).

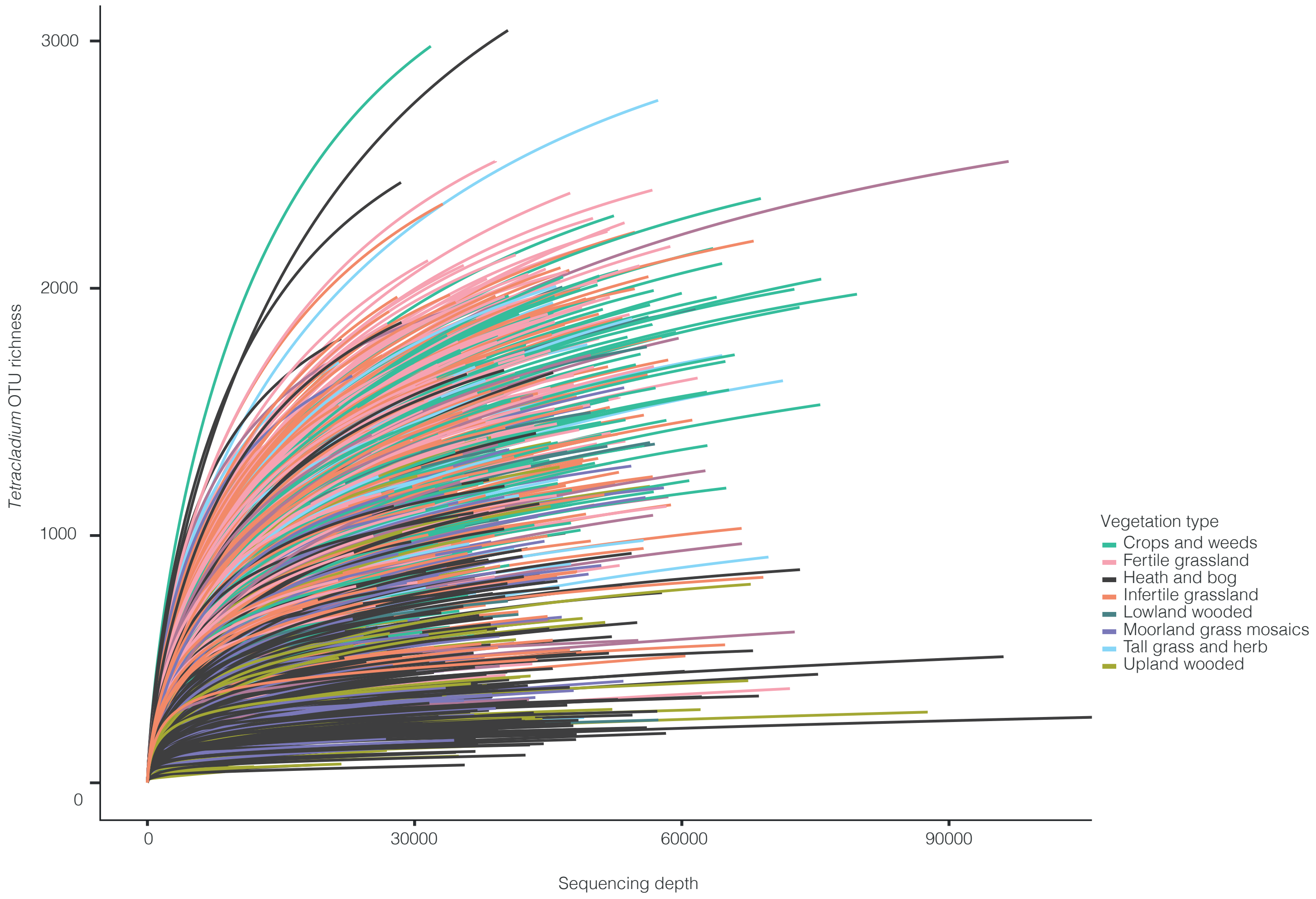

Supplementary Figure 2. Sequencing efficacy of the samples for the fungal ITS sequences. Rarefaction curves showing fungal OTU richness.

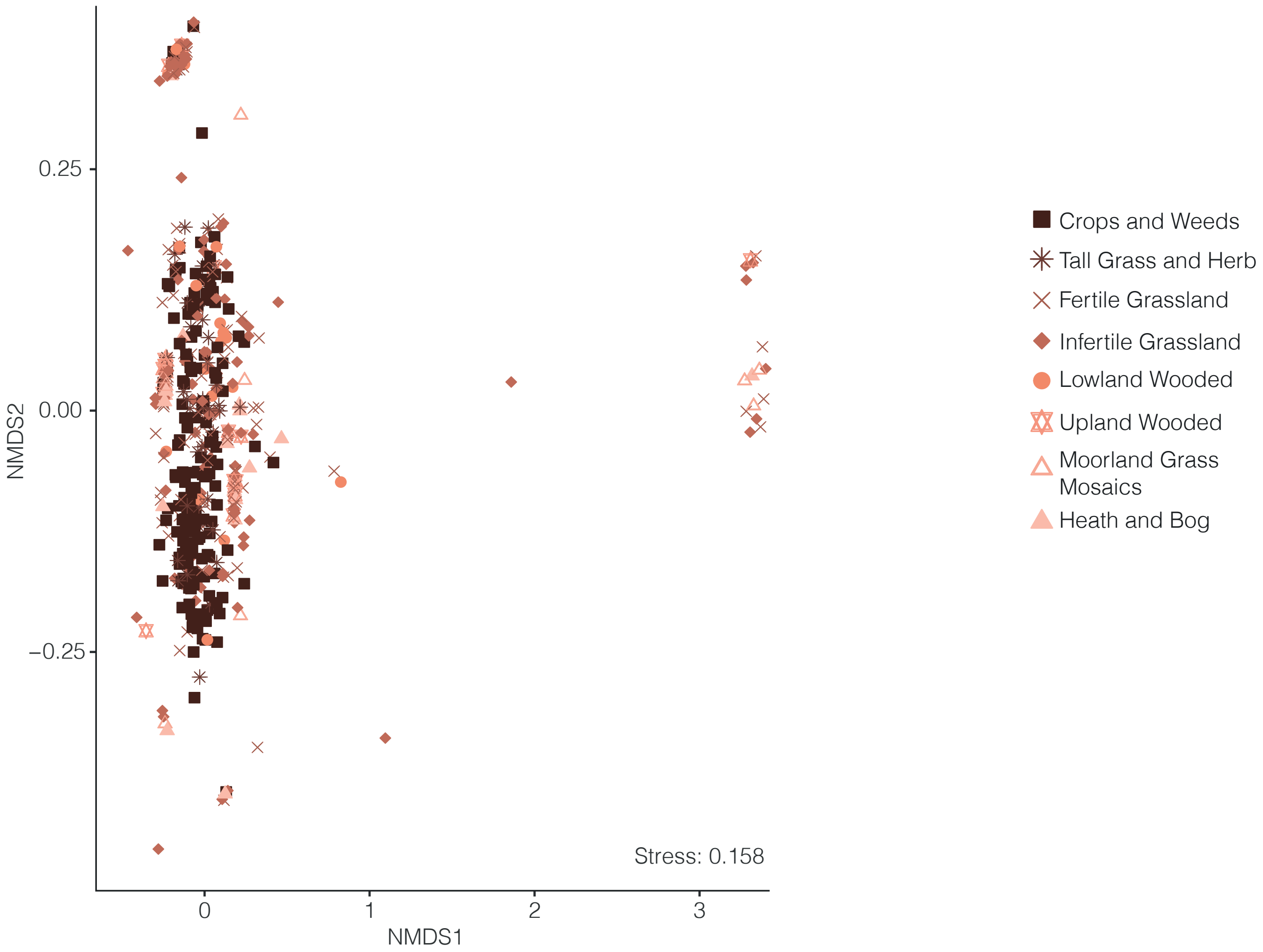

Supplementary Figure 3. Non-metric multidimensional scaling (NMDS) of the *Tetracladium* OTU community based on Raup-Crick dissimilarity of the vegetation types. Vegetation type colour denotes disturbance level (highly disturbed to natural habitats are shaded from dark to light).

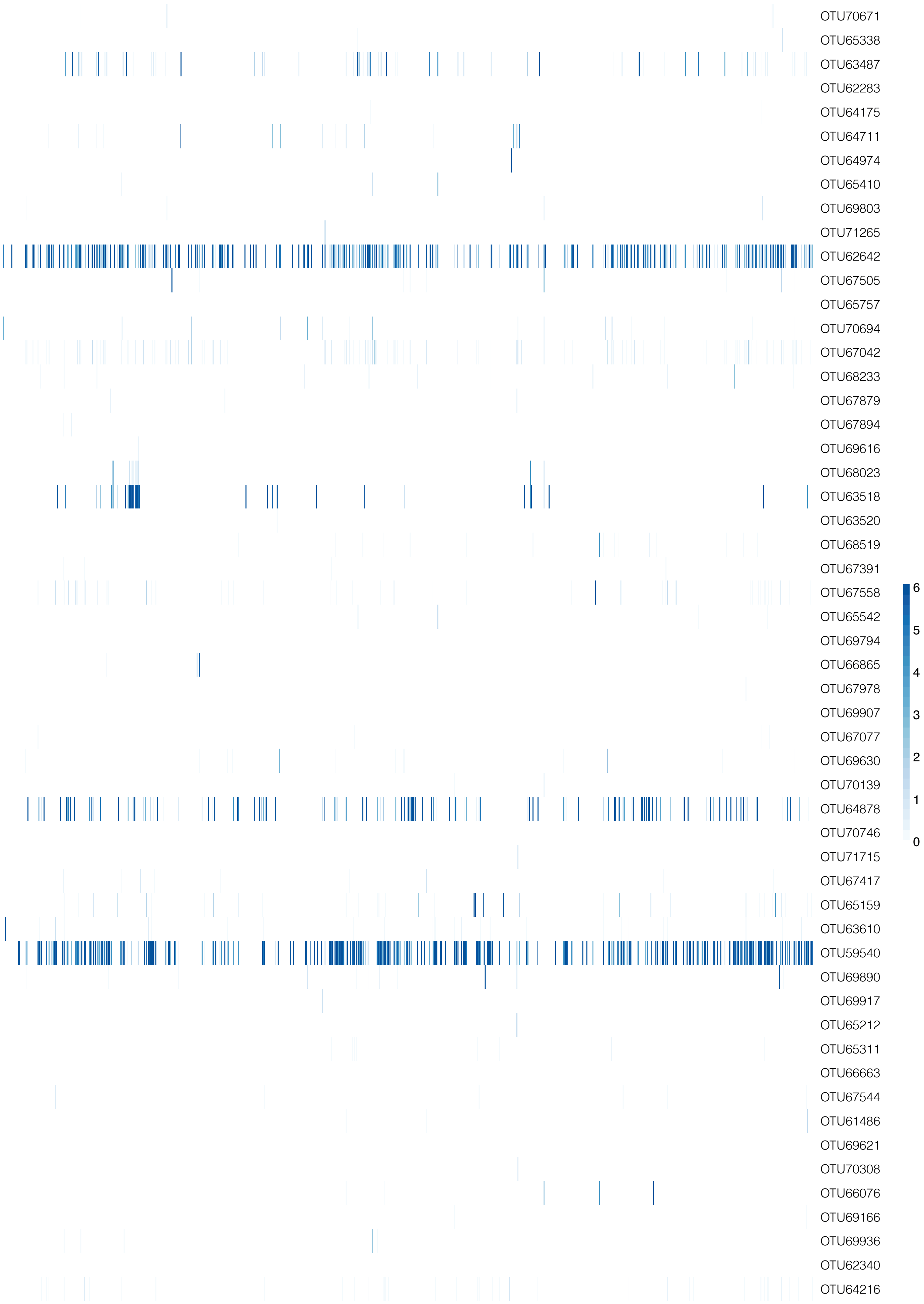

Supplementary Figure 4. Heatmap showing the distribution of *Tetracladium* OTUs across all samples.

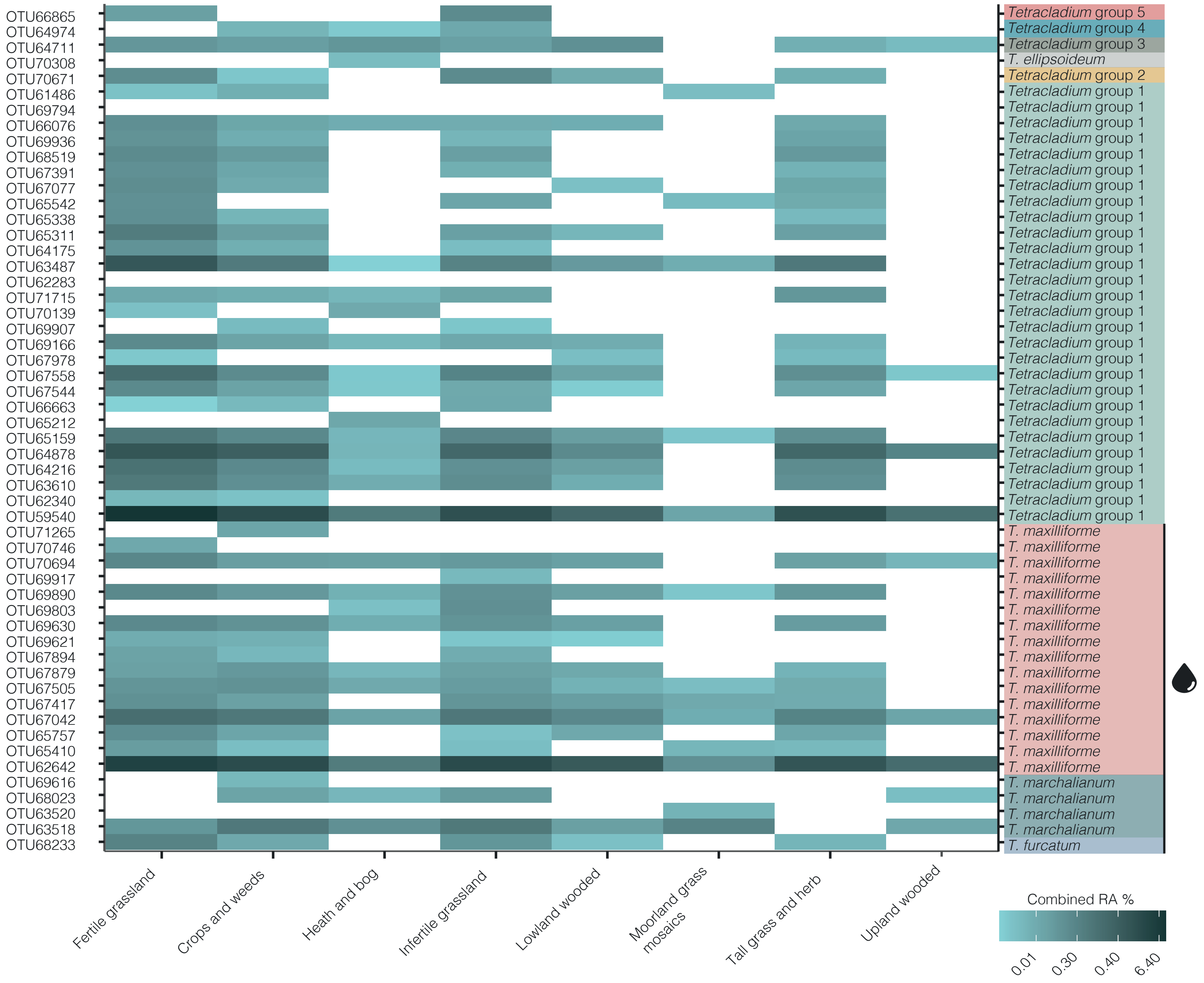

Supplementary Figure 5. Distribution of the OTUs across the vegetation types. Heatmap showing the combined relative abundance (RA) percent of *Tetracladium* OTUs across the vegetation types. The colours on the right side of the figure indicate groups from Figure 2. Taxa with water droplets next to them are traditionally considered aquatic.

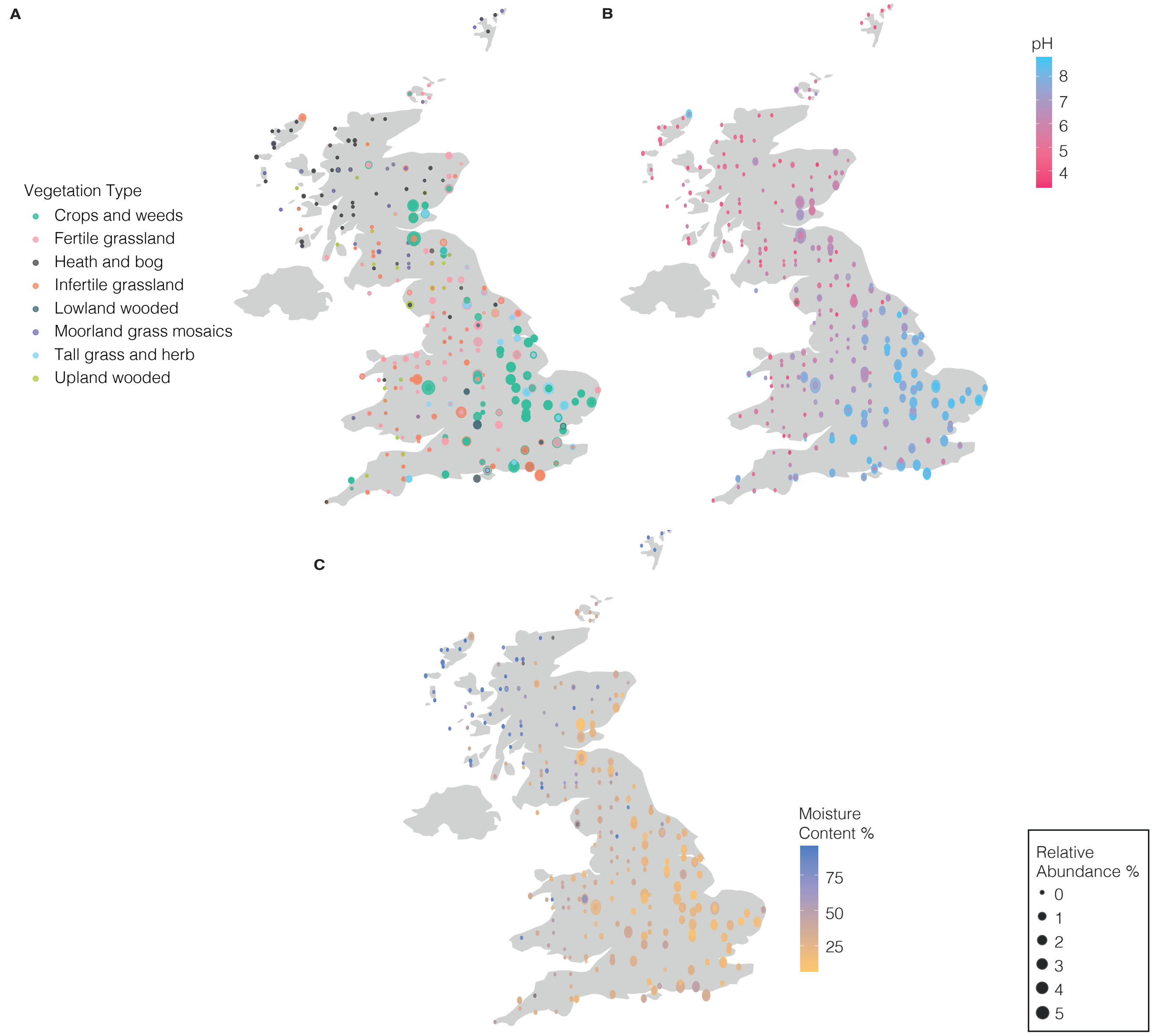

Supplementary Figure 6. Maps showing the location of the sampling sites, their A – vegetation type classifications, B – soil pH, C – soil moisture content (circle colour), and the combined *Tetracladium* OTU relative abundance percent (circle size).

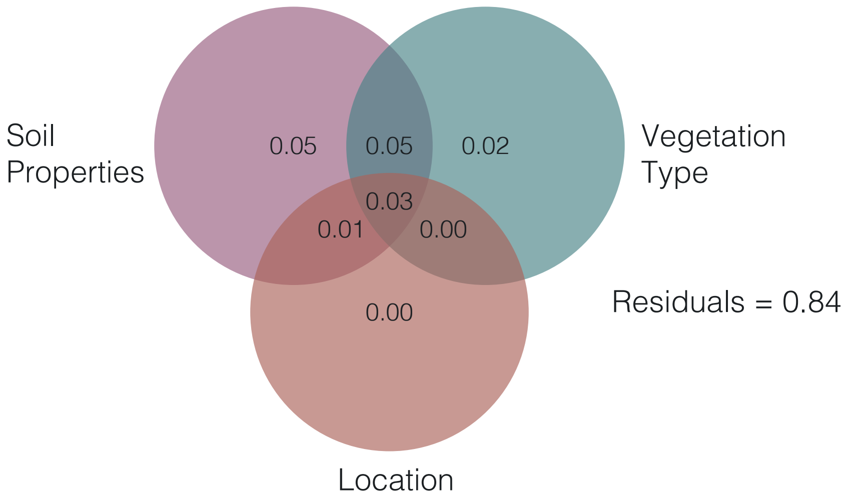

Supplementary Figure 7. Redundancy analysis of the variables shaping OTU richness. Venn diagram of the variation partitioned variable categories.

Supplementary Table 1. Brief description of the aggregate vegetation classes.

Aggregate vegetation class	Description
Crops and Weeds	This class occurs almost exclusively in fields on cultivated soils. It is quite common and has wheat as the main cover. It is very poor in plants with no one characteristic species.
Lowland Wooded	This class is virtually restricted to hedgerows or boundaries, but occasionally can be found by other linear features, usually between crops on brown soils.
Tall Grassland and Herb	This class occurs mainly in boundaries or by hedges between crops but occasionally beside grassland, small patches or other linear features on brown soils.
Fertile Grassland	This class occurs in all the plot types, except hedges, but it is especially common on roadsides and is usually on brown soils.
Infertile Grassland	This class is representive of the most widespread ordinary grassland type in Britain and is mainly present in fields, and is also be present by roads and occasionally elsewhere.
Upland Wooded	This class has many woodland species and occurs in a range of landscape elements containing trees, including hedges, on mildly acidic soils.
Moorland Grass Mosaics	This class usually occurs in small flushes but may be found in open vegetation and by streamsides on water-affected soils. Although usually in open vegetation, the class is also present besides roads or streams on podzolic or gleyed soils.
Heath and Bog	This class is often found on streamsides but is also common in open vegetation usually on gleyed soils.
